# A global survey to understand general vaccine trust, COVID-19 and influenza vaccine confidence

**DOI:** 10.3389/fpubh.2024.1406861

**Published:** 2024-11-20

**Authors:** Chelsea D’Silva, Madison M. Fullerton, Jia Hu, Kenneth Rabin, Scott C. Ratzan

**Affiliations:** ^1^19 to Zero Inc, Calgary, AB, Canada; ^2^Community Health Sciences, University of Calgary, Calgary, AB, Canada; ^3^School of Public Health and Health Policy, The City University of New York, New York, NY, United States

**Keywords:** vaccine trust, COVID-19, vaccine confidence, immunization, mRNA

## Abstract

**Introduction:**

The COVID-19 pandemic has greatly impacted the way that the world views vaccines. While safe and effective, COVID-19 vaccines were, and continue to be met with hesitancy and misinformation. We aimed to understand public perceptions and trust in COVID-19 vaccinations and how the pandemic has impacted perceptions of non-COVID-19 vaccines.

**Methods:**

Survey data were collected between August 7, 2023–August 16, 2023, from 7,000 respondents aged 18 years and older from the United States (*n* = 1,000); Nigeria (*n* = 1,000); United Kingdom (*n* = 1,000); France (*n* = 1,000); Canada (*n* = 1,000); Brazil (*n* = 1,000); and India (*n* = 1,000).

**Results:**

Trust in COVID-19 vaccines was highest in Brazil (84.6%) and India (80.4%) and lowest in the United States (63.5%) and France (55.0%). 47.5% of respondents agreed that they trust traditional protein-based vaccines more than mRNA vaccines, 13.5% disagree and 39.0% are neutral about their trust in protein-based versus mRNA vaccines. Overall, 53.9% of respondents reported that the COVID-19 pandemic impacted their perceptions of vaccines with half of these respondents (51.7%) reporting that the pandemic made them think that other vaccines are more important as they understand how critical vaccines can be at preventing serious illnesses.

**Discussion:**

These data can be used by health system decision makers, public health and researchers to understand how vaccine trust impacts perceptions of COVID-19 and influenza vaccines globally and develop tailored interventions that address local concerns.

## 1 Introduction

Public trust in the safety and efficacy of vaccines is essential to the success of immunization programs globally (1). Trust is often described as the key influence on vaccine acceptance (2) and it impacts not only personal health outcomes, but also the broader landscape of public health (2, 3). Vaccine trust extends beyond individual confidence in the safety and efficacy of a vaccine; rather it includes trust in the institutions that oversee its development, regulation, and administration. The interplay between perceived vaccine quality and safety, coupled with the credibility of the institutions endorsing the vaccine, significantly impact an individual’s likelihood of receiving a vaccine (2). A strong foundation of trust can bolster vaccine uptake, contributing to the achievement of herd immunity and the prevention of widespread infectious diseases. Interpersonal trust refers to the confidence an individual has in those directly responsible for communicating about and administering the vaccine. Personal characteristics including race, socioeconomic status, level of education, and religion profoundly affect interpersonal trust. These attributes influence who an individual interacts with to obtain information regarding vaccines and further shapes their views. This often results in increased interaction with those who validate their own perspectives (2). Trust in health care providers and trust in government confidence are strong drivers of vaccine acceptance across multiple countries and regions (4).

The global landscape of vaccine trust is characterized by a myriad of factors, including cultural, socioeconomic, political, and historical influences (5). Each country presents a unique set of circumstances that can either foster or challenge public confidence in vaccination efforts. Understanding these nuances can help tailor public health communication. Previous COVID-19 global surveys have shown large variation in vaccine acceptance across countries ranging from 47.9% in South Africa to 98.3% in India (6). Despite the disproportionate challenges in vaccine availability and distribution faced by low- and middle-income countries (LMICs), these countries tend to exhibit lower levels of vaccine hesitancy and higher acceptance rates than higher-income countries (7). Previous research shows that perceived susceptibility to COVID-19 infection, severity of complications, and believed benefit are associated with a higher intention to vaccinate (8). Meanwhile, people with concerns about the efficacy and side effects of COVID-19 vaccines are less likely to have a positive vaccination intent (8). As the world grapples with the challenges posed by COVID-19, understanding the dynamics of vaccine trust becomes paramount, not only for this virus but also in shaping broader attitudes toward other respiratory vaccinations, such as influenza vaccines. A recent review indicated that COVID-19 has increased intention to get influenza vaccinations (9). However, there are also reports of decreased influenza vaccination in healthcare personnel throughout the COVID-19 pandemic which is hypothesized to be due to COVID-19 vaccination campaigns leading to less emphasis on influenza vaccination or vaccine fatigue (10). Investigating the interconnectedness of vaccine trust and its repercussions on broader immunization initiatives can shed light on the potential ripple effects of building or eroding public trust.

Various research indicates that trust is integral to vaccine confidence; however, what type of trust has been up for debate. Trust in experts, scientists, medical authorities and medical professionals appears to have a small to moderate effect (11–13). Trust in government shows variation in the effect with a 25-sample study finding non-significant effects on vaccine confidence (13). Interestingly, a 19-country study (14) and an 8-country study (12) found significant effects of trust in government on vaccine acceptance. Finally, Rozek et al.’s (15) 17 country survey, found that trust in health institutions is significant but no effect for trust in political leaders.

The Vaccine Trust Gauge was developed from a previous scoping review (5) to create a standardized approach to measuring trust in vaccines (4). This validated and reliable tool includes perception of vaccine safety, efficacy, and importance, while also inquiring about trust in information sources (16). This paper uses the vaccine trust gauge to delve into the intricate interplay of vaccine trust on a global scale, with a specific focus on COVID-19 and influenza vaccines. By examining patterns of trust across different countries, including Canada, Brazil, France, India, the United States, the United Kingdom, and Nigeria, we aim to unravel the factors influencing public perception.

## 2 Methods

We conducted an observational cross-sectional survey to explore how vaccine trust differs across countries and the relationship between overall vaccine trust and perceptions of COVID-19 and influenza vaccines.

### 2.1 Survey instruments

The survey instrument contained 4 parts: (1) demographic questions; (2) the Vaccine Trust Gauge (4); (3) COVID-19 vaccine related questions and (4) Influenza vaccines-related questions. Demographic questions included sex, age, education, and average yearly income. The Vaccine Trust Gauge is a series of questions that measure overall vaccine trust levels and has a high internal reliability (Cronbach’s alpha =0.947) (16). Additional questions related to perceptions about COVID-19 and influenza vaccines were also included in the survey instrument based on the recommendations of public health and infectious disease physicians. These questions were adapted from previous studies and focused on the knowledge, perceived safety and efficacy and intention to receive COVID-19 and influenza vaccinations (6, 14, 17). The full survey instrument can be found in the Supplementary material.

### 2.2 Recruitment and data collection

Survey data were collected between 7 August - 16 August 2023 from *N* = 7,000 respondents aged 18 years and older from the United States (*n* = 1,000); Nigeria (*n* = 1,000); United Kingdom (*n* = 1,000); France (*n* = 1,000); Canada (*n* = 1,000); Brazil (*n* = 1,000); and India (*n* = 1,000). An online opt-in panel of participants was provided by Consensus Strategies and participants were recruited by telephone contact, social media outreach and direct email solicitation. Social media outreach was complete by posting recruitment materials on social media platforms (X and Instagram) where participants were directed toward the online survey. The online survey was available in English, French, Portuguese and Hindi based on the predominant languages in each country. A stratum-based sample design was implemented based on age, gender, statistical regions, median income, and levels of education for each country; a minimum of 50 participants was set for each stratum, with target enrollment calculated to reflect the distribution of each subgroup in the general population of each country. This survey was administered by Emerson College, located in Boston, U.S.A. No personally identifiable information was collected or stored. This approach has been utilized in previous literature to recruit a random sample of the population (6). This project was reviewed and deemed exempt from research by Emerson College’s Institutional Review Board (protocol number 22-019-F-X).

### 2.3 Analysis

Descriptive statistics were calculated for all variables. Similar to previous papers using the Vaccine Trust Gauge (4), scores from the Vaccine Trust Gauge survey questions were aggregated and then converted to a 0.0–1.0 scale with 0.0 representing no trust at all and 1.0 representing complete trust. The Vaccine Trust Gauge scores were then categorized into high, medium and low trust levels using 0.33 intervals. Multinomial logistic regressions were conducted to explore the association between demographic characteristics, COVID-19 vaccine perspectives and influenza vaccine perspectives and vaccine trust levels. Responses for COVID-19 and influenza vaccine perspectives were categorized as agree (strongly agree and somewhat agree), neutral, and disagree (strongly disagree and somewhat disagree). All analyses were conducted in SAS version 9.4 software.

## 3 Results

### 3.1 Demographics

A total of 7,000 people responded to the survey including 1,000 people from Brazil, Canada, France, India, Nigeria, the United Kingdom, and the United States. Women comprised 50.1% of the study population, and 50.0% of all participants earned less than the average median income while 50% earned above the median income. One in five participants had a university degree. Respondent characteristics by country are listed in Table 1.

**Table 1:** Participant demographics by country.

	Total	Brazil	Canada	France	India	Nigeria	U.K.	U.S.
	*n* = 7,000	*n* = 1,000	*n* = 1,000	*n* = 1,000	*n* = 1,000	*n* = 1,000	*n* = 1,000	n = 1,000
Sex								
Female	50.1%	50.5%	50.0%	51.0%	49.5%	49.5%	50.0%	50.2%
Male	49.4%	49.4%	49.0%	48.5%	50.5%	50.5%	49.3%	48.9%
Non-binary	0.5%	0.1%	1.0%	0.5%	0.0%	0.0%	0.7%	0.9%
Age								
18–24	15.3%	17.8%	9.7%	10.5%	17.6%	28.4%	11.2%	11.7%
25–34	19.7%	22.4%	18.0%	15.2%	23.0%	23.4%	17.5%	18.1%
35–44	18.4%	17.4%	16.9%	16.5%	24.0%	21.5%	15.8%	16.8%
45–54	14.8%	16.2%	15.1%	14.7%	14.8%	13.1%	14.5%	15.5%
55–64	14.1%	13.5%	17.1%	16.4%	11.1%	7.5%	16.7%	16.7%
65 or older	17.6%	12.6%	23.1%	26.8%	9.4%	6.2%	24.3%	21.1%
Education								
No college degree	78.7%	83.0%	73.6%	82.1%	91.0%	91.4%	66.1%	64.0%
College degree or more	21.3%	17.0%	26.4%	17.9%	9.0%	8.6%	33.9%	36.0%
Average yearly income								
Below average	50.0%	50.0%	50.0%	50.0%	50.0%	50.0%	50.0%	50.0%
Above average	50.0%	50.0%	50.0%	50.0%	50.0%	50.0%	50.0%	50.0%
Healthcare worker?								
Yes	6.8%	3.9%	6.9%	8.5%	6.6%	8.8%	7.3%	5.9%
No	93.2%	96.1%	93.1%	91.5%	93.4%	91.2%	92.7%	94.1%

Multinomial logistic regression showed the association between demographic characteristics and vaccine trust levels. The odds of having high vaccine trust decrease by 1.4% for every one-year increase in age (*p* < 0.001). Having no college education significantly decreases the odds of having high vaccine trust by 49.5% (*p* < 0.001). Having below-average yearly income significantly decreases the odds of having high vaccine trust by 49.5% (*p* < 0.001). The odds ratios (OR) and 95% confidence intervals (CI) for each predictor variable are presented in Table 2.

**Table 2:** Participant demographics associated with vaccine trust levels.

	Total	Brazil	Canada	India	Nigeria	UK
	OR (95% CI)	OR (95% CI)	OR (95% CI)	OR (95% CI)	OR (95% CI)	OR (95% CI)
Medium vaccine trust						
Age	0.981 (0.975–0.986) ***	0.989 (0.966–1.012)	0.983 (0.970–0.997)	1.019 (0.977–1.063)	0.996 (0.962–1.031)	0.971 (0.957–0.985)***
Gender: female *vs* male	1.111 (0.904–1.366)	1.637 (0.772–3.470)	1.631 (1.009–2.636)	2.065 (0.505–3.625)	0.534 (0.128–2.237)	0.946 (0.563–1.591)
Education: no college *vs* college	0.743 (0.545–1.013)	0.501 (0.120–2.090)	0.633 (0.321 1.249)	2.412(0.140–4.684)	0.937 (0.058–1.820)	0.575 (0.292–1.129)
Yearly income: below average *vs* above average	0.684 (0.551–0.849) ***	0.401 (0.177–0.909)	0.900 (0.549–1.475)	0.03 (0.001–0.978)*	0.037 (0.001–1.295)	1.636 (0.965–2.774)
High vaccine trust						
Age	0.986 (0.981–0.991)***	0.997 (0.975–1.018)	1.005 (0.992–1.018)	1.026 (0.983–1.070)	0.9670.9341.002	0.995 (0.982–1.009)
Gender: female *vs* male	0.959 (0.787–1.169)	2.347 (1.164–4.732)*	1.202 (0.761–1.897)	3.188 (0.787–5.589)	0.3320.081.385	0.695 (0.426–1.134)
Education: no college *vs* college	0.505 (0.375–0.679)***	0.359 (0.091–1.412)	0.378 (0.198–0.721)**	1.630 (0.096–3.164)	0.2750.0174.415	0.272 (0.144–0.514)***
Yearly income: Below average *vs* above average	0.505 (0.411–0.622)***	0.301 (0.140–0.651)**	0.609 (0.382–0.972)	0.027 (0.001–0.869)*	0.0290.0011.008	1.218 (0.741–2.003)

*p < 0.01; **p < 0.005; ***p < 0.001.

### 3.2 Vaccine trust levels by country

**Figure 1:**
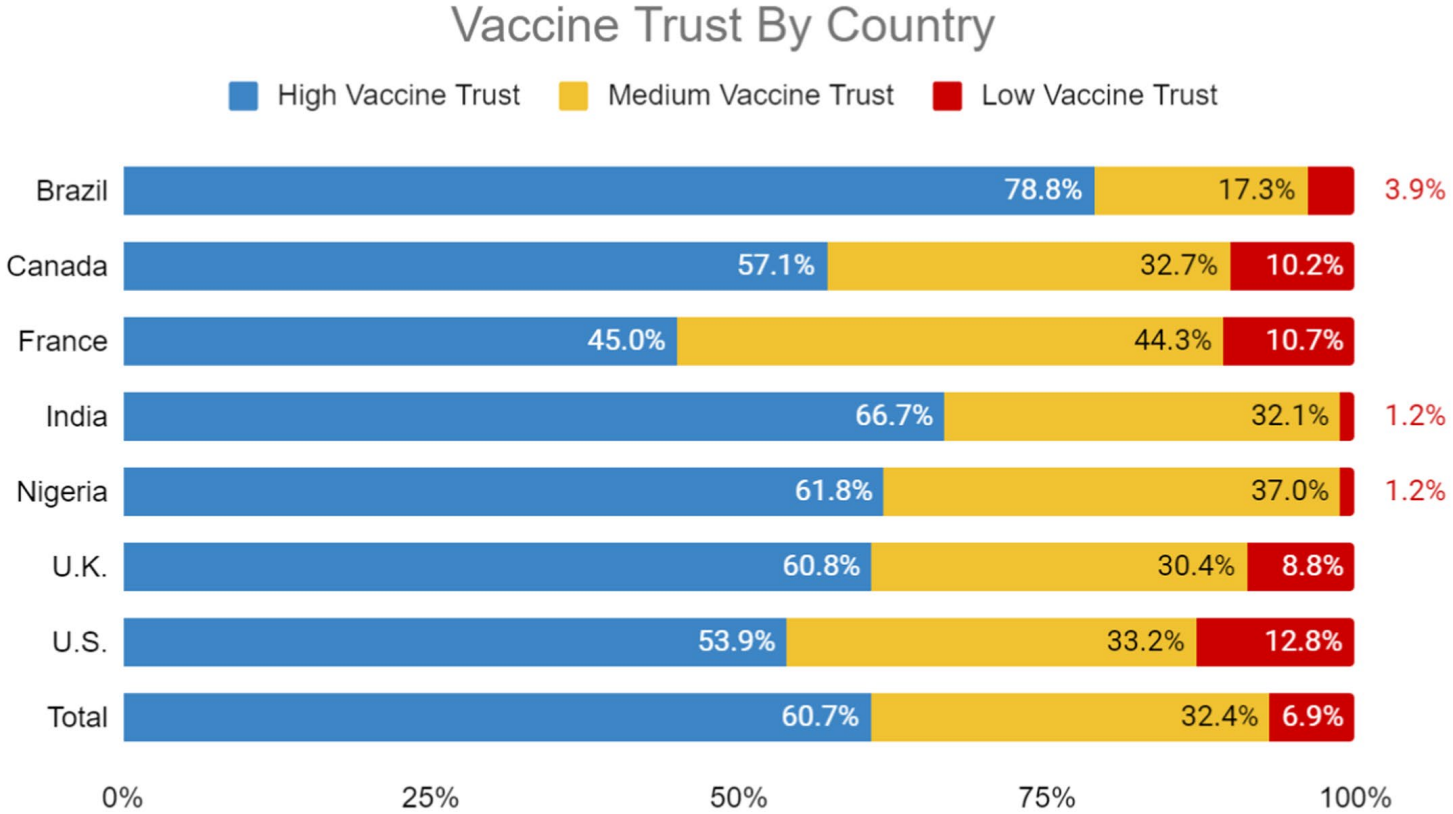
Vaccine trust by country.

Brazil (78.8%), India (66.7%), Nigeria (61.8%) and the UK (60.8%) had the highest proportion of participants with high vaccine trust. The United States (12.8%), France (10.7%), Canada (10.2%) and the UK (8.8%) had the highest proportion of participants with low vaccine trust (Figure 1).

### 3.3 COVID-19 vaccine perceptions

**Figure 2:**
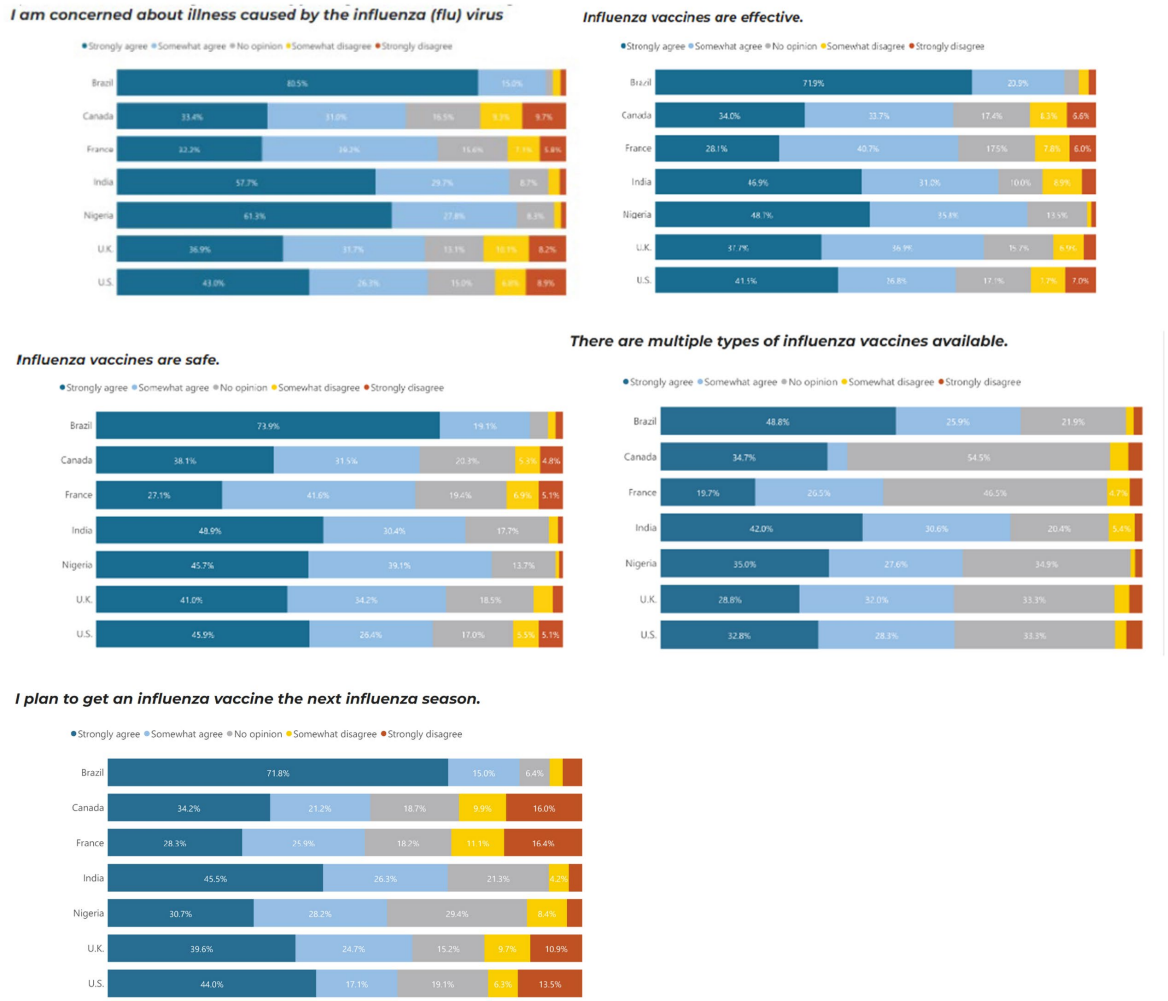
COVID-19 vaccine perceptions by country.

Overall, Brazil (82.9%), Nigeria (68.6%) and India (56.8%) were the countries most concerned about illnesses caused by COVID-19. They were also the countries with the most trust in the safety, efficacy and science behind COVID-19 vaccines. Brazil and India had the highest proportion of people who would continue to receive additional booster vaccines and reported the importance of ensuring the booster matches the current strain of COVID-19. 47.5% of respondents agreed that they trust traditional protein-based vaccines more than mRNA vaccines, 13.5% disagree and 39.0% are neutral about their trust in protein-based versus mRNA vaccines (Figure 2).

India (80.2%), Brazil (71.6%) and Nigeria (67.8%) were the countries that most reported that the pandemic and their knowledge of COVID-19 vaccines changed their perspectives on other vaccines. Of those that said the pandemic impacted their perception of vaccines, increased concerns about the efficacy (72.7%) and safety (68.2%) of vaccines were the main impact of the pandemic on vaccine perceptions.

Table 3 shows the association between vaccine trust levels and COVID-19 perspectives. Those with high vaccine trust were more likely to agree with COVID-19 vaccine confidence sentiments regardless of country. Participants who were concerned about illness caused by COVID-19 had 2.5 times higher odds of having high vaccine trust compared to those who disagreed (*p* < 0.001). Participants who believe that COVID-19 vaccines are effective had 7.4 times higher odds of having high vaccine trust compared to those who disagreed (*p* < 0.001). Participants who agreed that COVID-19 vaccines are safe had 5.2 times higher odds of having high vaccine trust compared to those who disagreed (*p* < 0.001). Participants who agreed that they trust the science behind COVID-19 vaccines had 5.2 times higher odds of having high vaccine trust compared to those who disagreed (*p* < 0.001). Participants who agreed that they trust traditional vaccines more than mRNA vaccines had 17.2 times higher odds of having high vaccine trust compared to those who disagreed (*p* < 0.001).

**Table 3:** Association between vaccine trust levels and COVID-19 perspectives.

	Total	Brazil	Canada	France	India	Nigeria	UK	US
	OR (95% CI)	OR (95% CI)	OR (95% CI)	OR (95% CI)	OR (95% CI)	OR (95% CI)	OR (95% CI)	OR (95% CI)
High vaccine trust								
I am concerned about illness caused by COVID-19: agree *vs* disagree	2.456 (1.696–3.557)***		0.685 (0.274–1.713)	2.874 (1.085–7.612)	0.246 (0.005–1.569)	2.336 (0.313–11.424)	1.034 (0.391–2.734)	5.89 (2.556–13.569)***
I am concerned about illness caused by COVID-19: neutral *vs* disagree	1.111 (0.667–1.852)		0.578 (0.131–2.546)	0.688 (0.207–2.291)	0.935 (0.008–1.003)	0.986 (0.035–2.994)	1.245 (0.306–5.069)	1.738 (0.563–5.368)
COVID-19 vaccines are effective: agree *vs* disagree	7.361 (3.599–11.123)***	7.536 (1.236–13.936)	8.500 (1.355–15.531)	9.340 (1.589–15.904)	7.536 (1.236–13.936)	3.934 (0.130–6.307)	0.986 (0.035–2.994)***	1.790 (0.387–8.291)
COVID-19 vaccines are effective: neutral *vs* disagree	1.249 (0.732–2.13)	0.437 (0.0610–3.140)	2.026 (0.473–8.679)	2.416 (0.715–8.165)	4.253 (0.0161–9.210)	0.102 (0.013–0.790)	6.47 (1.562–6.831)*	0.387 (0.098–1.535)
COVID-19 vaccines are safe: agree *vs* disagree	5.221 (2.112–8.330)***	5.065 (0.740–10.069)	1.441 (0.213–9.769)	2.066 (1.821–2.519)	1.361 (0.007–3.770)	4.892 (0.149–8.946)	4.653 (0.527–9.101)	8.825 (1.688–19.775)
COVID-19 vaccines are safe: neutral *vs* disagree	0.931 (0.538–1.612)	2.567 (0.325–4.809)	1.466 (0.338–6.355)	0.716 (0.179–2.859)	1.771 (0.003–3.175)	6.851 (0.436–12.696)	0.901 (0.206–3.933)	0.643 (0.159–2.601)
I trust the science behind the COVID-19 vaccines: agree *vs* disagree	17.275 (6.846–27.704)***	1.409 (0.298–6.657)	1.834 (0.733–2.965)	3.561 (1.729–7.042)	0.563 (0.003–1.838)	0.199 (0.005–1.249)	6.239 (2.735–12.420)**	3.635 (1.162–7.288)
I trust the science behind the COVID-19 vaccines: neutral *vs* disagree	2.703 (1.548–4.72)***	0.874 (0.158–4.843)	4.261 (0.969–9.736)	2.616 (0.718–9.525)	0.886 (0.007–1.242)	0.821 (0.076–1.933)	4.341 (0.820–8.557)	2.675 (0.645–11.097)
I will continue to get boosted for COVID-19 vaccine if it is recommended to me: agree *vs* disagree	4.232 (1.946–9.207)***	8.433 (1.596–15.270)	1.137 (0.937–2.389)	1.253 (0.289–5.433)	1.253 (0.289–5.433)	2.221 (0.574–4.682)	1.518 (0.231–3 0.951)	4.313 (2.464–9.931)*
I will continue to get boosted for COVID-19 vaccine if it is recommended to me: neutral *vs* disagree	1.163 (0.674–2.007)	0.189 (0.034–1.051)	1.249 (0.331–4.720)	4.988 (1.223–8.337)	4.988 (1.223–8.337)	1.919 (0.653–2.869)	0.273 (0.05–1.488)	2.238 (0.677–7.394)
It is important that any booster vaccine I get matches the current circulating variant(s): agree *vs* disagree	4.758 (2.825–8.012)***	0.265 (0.031–2.301)	2.276 (0.602–8.608)	7.105 (1.603–14.485)*	7.426 (0.057–14.957)	1.354 (0.201–9.100)	2.678 (0.618–5.612)	9.957 (2.183–16.407)**
It is important that any booster vaccine I get matches the current circulating variant(s): neutral *vs* disagree	1.468 (0.909–2.371)	0.177 (0.017–1.795)	1.021 (0.320–3.260)	6.591 (1.646–12.391)*	10.347 (0.293–19.443)	0.244 (0.031–1.897)	0.946 (0.244–3.664)	2.032 (0.523–7.899)
I trust traditional vaccines (e g, protein-based vaccines) more than mRNA vaccines: agree *vs* disagree	5.206 (3.503–7.739)***	3.471 (0.973–5.969)	1.401 (0.528–3.718)	5.12 (1.984–13.211)***	6.591 (1.646–12.391)*	5.960 (0.683–11.044)	2.912 (0.8421–0.075)	6.249 (2.242–12.417)***
I trust traditional vaccines (e g, protein-based vaccines) more than mRNA vaccines: neutral *vs* disagree	2.544 (1.736–3.73)***	3.203 (0.825–5.581)	1.068 (0.388–2.937)	2.364 (0.991–5.643)	2.364 (0.991–5.643)	7.980 (0.876–15.694)	1.023 (0.310–3.373)	9.471 (3.496–17.655)***
Medium vaccine trust								
I am concerned about illness caused by COVID-19: agree *vs* disagree	2.319 (1.714–3.137)***	2.331 (1.458–3.118)***	1.667 (0.809–3.436)	2.462 (1.177–5.149)	0.145 (0.003–7.007)	2.392 (0.384–4.910)	1.512 (0.666–3.433)	2.627 (1.38–5.001)**
I am concerned about illness caused by COVID-19: neutral *vs* disagree	1.735 (1.148–2.621)*	1.735 (1.148–2.621)	2.793 (0.767–5.173)	0.898 (0.357–2.259)	3.474 (0.035–6.518)	0.506 (0.022–1.897)	1.876 (0.556–6.332)	1.186 (0.510–2.759)
COVID-19 vaccines are effective: agree *vs* disagree	3.729 (1.938–7.176)***	1.970 (0.479–8.102)	6.076 (1.212–11.455)	11.136 (2.155–19.556)**	2.462 (1.177–5.149)	2.093 (0.077–5.138)	3.912 (2.157–5.075)	0.950 (0.270–3.344)
COVID-19 vaccines are effective: neutral *vs* disagree	1.265 (0.838–1.910)	0.767 (0.177–3.315)	2.477 (0.843–7.281)	2.860 (1.040–7.861)	6.690 (0.028–12.475)	0.084 (0.014–0.504)*	2.443 (0.843–7.079)	0.769 (0.296–1.998)
COVID-19 vaccines are safe: agree *vs* disagree	3.875 (1.651–9.09)**	2.502 (0.481–3.048)	0.375 (0.069–2.020)	2.477 (0.843–7.281)	6.892 (0.041–17.669)	5.760 (0.189–11.623)	5.783 (0.776–11.084)	2.398 (1.763–3.731)
COVID-19 vaccines are safe: neutral *vs* disagree	1.674 (1.097–2.557)	2.780 (0.565–4.995)	1.428 (0.479–4.256)	0.674 (0.203–2.235)	1.428 (0.479–4.256)	2.112 (1.103–3.083)	1.778 (0.56–5.651)	1.318 (0.552–3.147)
I trust the science behind the COVID-19 vaccines: agree *vs* disagree	5.016 (2.063–7.969)***	1.057 (0.273–4.100)	0.375 (0.069–2.020)	7.921 (0.614–10.113)	0.202 (0.001–0.362)	0.069 (0.002–2.688)	4.234 (0.831–8.562)	3.041 (0.391–6.624)
I trust the science behind the COVID-19 vaccines: neutral *vs* disagree	2.37 (1.495–3.755)***	1.106 (0.318–3.845)	2.7140 (0.882–8.349)	3.302 (1.095–9.963)	1.138 (0.011–2.446)	0.294 (0.03–2.843)	8.255 (1.926–15.386)**	1.531 (0.530–4.419)
I will continue to get boosted for COVID-19 vaccine if it is recommended to me: agree *vs* disagree	1.657 (0.785–3.497)	1.665 (0.405–6.850)	4.743 (0.423–6.196)	0.399 (0.104–1.535)	3.302 (1.095–9.963)	0.442 (0.172–1.098)	0.272 (0.046–1.61)	6.253 (0.677–12.784)
I will continue to get boosted for COVID-19 vaccine if it is recommended to me: neutral *vs* disagree	1.458 (0.908–2.341)	0.742 (0.219–2.509)	1.542 (0.487–4.883)	3.098 (0.883–6.861)	0.399 (0.104–1.535)	5.220 (0.666–10.847)	0.233 (0.051–1.058)	1.477 (0.549–3.975)
It is important that any booster vaccine I get matches the current circulating variant(s): agree *vs* disagree	2.378 (1.555–3.636)***	0.127 (0.027–0.591)**	1.950 (0.663–5.733)	1.800 (0.0.684–4.733)	0.932 (0.008–1.374)	0.768 (0.128–4.613)	4.496 (1.394–8.505)	2.108 (0.71–6.256)
It is important that any booster vaccine I get matches the current circulating variant(s): neutral *vs* disagree	1.393 (0.988–1.966)	0.128 (0.026–0.639)	0.921 (0.409–2.077)	1.099 (0.482–2.505)	1.467 (0.048–2.450)	0.719 (0.107–4.812)	2.201 (0.829–5.846)	0.828 (0.394–1.74)
I trust traditional vaccines (e g, protein-based vaccines) more than mRNA vaccines: agree *vs* disagree	3.670 (2.602–5.177)***	4.141 (1.085–7.197)	2.680 (1.238–5.803)	8.147 (3.582–18.526)***	0.921 (0.409–2.077)	2.763 (0.327–4.323)	3.705 (1.379–9.955)*	4.233 (1.960–9.144)***
I trust traditional vaccines (e g, protein-based vaccines) more than mRNA vaccines: neutral *vs* disagree	2.267 (1.638–3.139)***	3.507 (0.848–6.166)	2.28 (1.001–5.190)	4.406 (2.109–9.205)***	2.680 (1.238–5.803)	5.575 (0.631–10.235)	1.807 (0.721–4.526)	4.53 (2.208–9.293)***

*p < 0.01; **p < 0.005; ***p < 0.001.

### 3.4 Influenza vaccine perceptions

**Figure 3:**
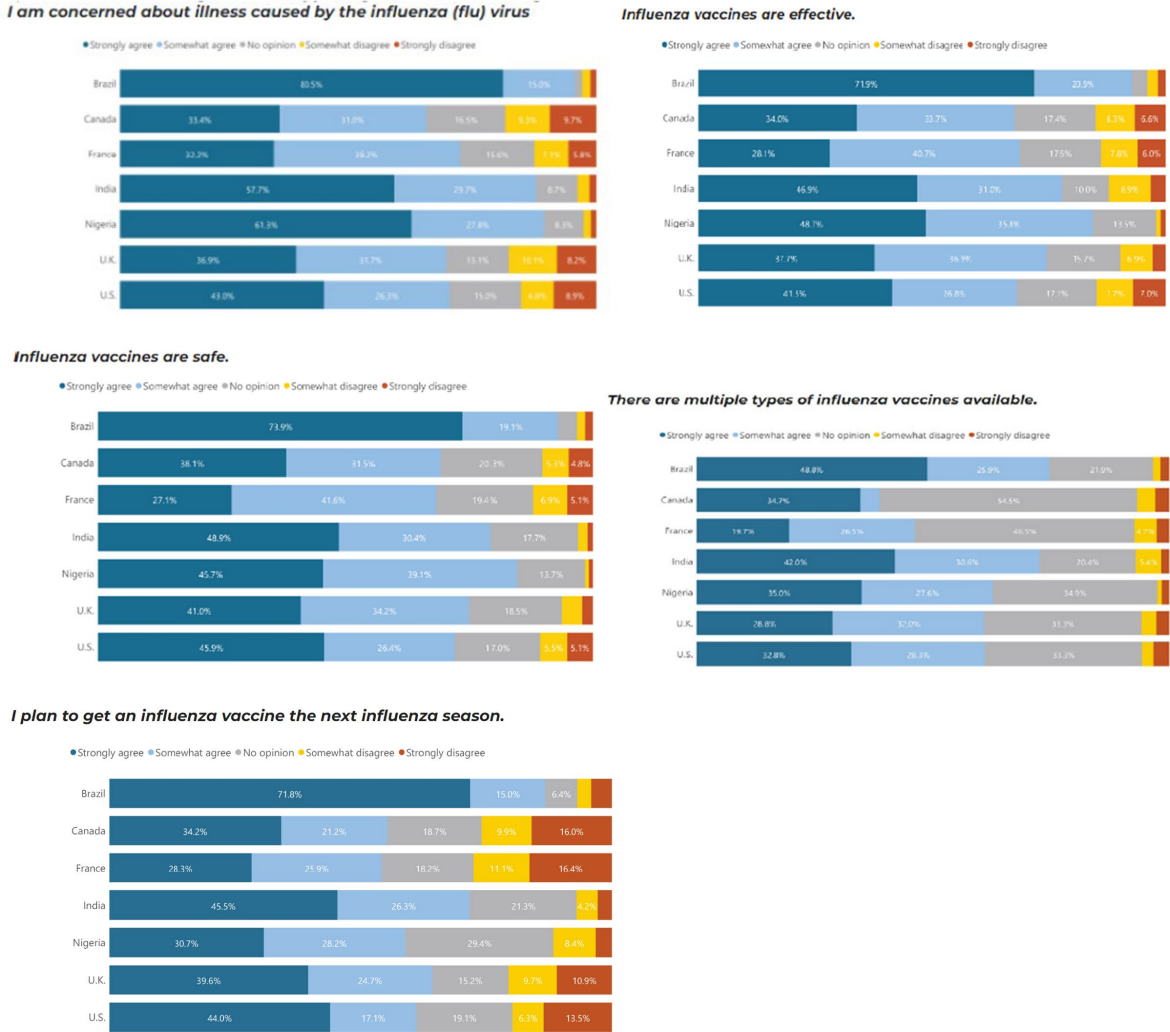
Influenza vaccine perceptions by country.

Similar to COVID-19, Brazil (80.5%), Nigeria (61.3%) and India (57.5%) were the countries most concerned about illnesses caused by influenza, and they were also the countries with the most trust in the safety, efficacy and science behind COVID-19 vaccines. In comparison, Brazil and Nigeria, the UK and the US were marginally more concerned about COVID-19 than influenza. Brazil and India had the highest proportion of people who would continue to receive additional booster vaccines and reported the importance of ensuring the booster matches the current strain of COVID-19 (Figure 3).

Those with high vaccine trust were more likely to agree with influenza vaccine confidence sentiments regardless of country. Individuals with a high level of concern about illness caused by the influenza virus have 1.9 times higher odds of having high vaccine trust compared to those with low vaccine trust (*p* = 0.002). Individuals who agree that influenza vaccines are effective have 3.5 times higher odds of having high vaccine trust compared to those with low vaccine trust (*p* < 0.001). Individuals who agree that influenza vaccines are safe have 18.7 times higher odds of having high vaccine trust compared to those with low vaccine trust (*p* < 0.001). Individuals who agree that they plan to get an influenza vaccine in the next season have significantly higher odds of having high vaccine trust compared to those who disagree (*p* < 0.001) (Table 4).

**Table 4:** Influenza vaccine perceptions by country.

	Total	Brazil	Canada	France	India	Nigeria	UK	US
	OR (95% CI)	OR (95% CI)	OR (95% CI)	OR (95% CI)	OR (95% CI)	OR (95% CI)	OR (95% CI)	OR (95% CI)
High vaccine trust								
I am concerned about illness caused by influenza: agree *vs* disagree	1.858 (1.264–2.73)**	5.545 (0.649–9.388)	0.644 (0.257–1.618)	1.537 (0.598–3.953)	1.537 (0.598–3.953)	1.728 (0.201–2.861)	2.86 (1.238–6.607)	1.924 (0.816–4.534)
I am concerned about illness caused by influenza: neutral *vs* disagree	0.858 (0.552–1.334)	2.307 (0.099–4.518)	1.56 (0.526–4.626)	0.721 (0.273–1.903)	0.721 (0.273–1.903)	25.441 (0.365–38.826)	2.139 (0.804–5.692)	0.388 (0.133–1.136)
Influenza vaccines are effective: agree *vs* disagree	3.483 (2.141–5.665)***	5.211 (0.446–10.904)	1.617 (0.468–5.583)	2.428 (0.762–7.732)	2.428 (0.762–7.732)	1.339 (0.04–2.457)	4.066 (1.276–8.958)	9.27 (2.097–16.984)**
Influenza vaccines are effective: neutral *vs* disagree	1.637 (1.041–2.573)	1.085 (0.109–1.788)	1.1 (0.388–3.119)	1.079 (0.384–3.026)	1.079 (0.384–3.026)	0.484 (0.014–2.216)	2.356 (0.754–7.358)	3.612 (0.817–6.964)
Influenza vaccines are safe: agree *vs* disagree	18.714 (10.605–26.026)***	1.531 (0.051–3.57)	3.84 (0.1795–6.857)***	6.529 (3.872–9.551)***	4.529 (1.872–7.551)***	4.009 (0.043–7.351)	3.84 (0.1795–6.857)***	8.391 (2.861–12.605)***
Influenza vaccines are safe: neutral *vs* disagree	2.842 (1.688–4.785)***	1.23 (0.049–5.946)	3.849 (1.022–6.489)	3.543 (1.176–10.677)	3.543 (1.176–6.677)	2.089 (0.029–4.56)	1.861 (0.498–6.953)	3.214 (0.559–6.466)
There are multiple types of influenza vaccines available: agree *vs* disagree	5.996 (3.798–9.467)***	19.341 (2.936–27.39)	9.966 (5.714–15.929)***	10.462 (3.764–17.076)***	10.462 (3.764–17.076)***	0.86 (0.017–1.028)	2.312 (0.924–5.783)	5.463 (1.307–7.826)
There are multiple types of influenza vaccines available: neutral *vs* disagree	3.141 (1.941–5.083)***	2.666 (0.177–5.136)	8.404 (2.45–16.826)***	4.623 (1.572–13.6)*	4.623 (1.572–7.6)*	1.306 (0.023–2.558)	1.547 (0.522–4.59)	1.898 (0.397–4.078)
I plan to get an influenza vaccine the next influenza season: agree *vs* disagree	8.21(5.361–12.574)***	9.542(6.637–12.742)	9.318(2.96–17.336)***	1.47(0.5483.944)	1.47(0.548–3.944)	6.488(5.921–7.118)***	8.391(2.861–12.605)***	6.837(2.569–11.196)***
I plan to get an influenza vaccine the next influenza season: neutral *vs* disagree	1.941 (1.349–2.794)***	4.021 (0.629–8.688)	5.757 (2.051–10.163)***	0.952 (0.39–2.32)	0.952 (0.39–2.32)	10.171* (2.051–18.442)	1.259 (0.526–3.011)	2.171 (0.896–5.256)
Medium vaccine trust								
I am concerned about illness caused by influenza: agree *vs* disagree	2.031 (1.444–2.857)***	7.617 (1.564–14.103)	0.86 (0.384–1.928)	2.218 (0.962–5.115)	2.218 (0.962–5.115)	2.299 (0.274–4.285)	2.517 (1.162–5.452)	1.091 (0.512–2.322)
I am concerned about illness caused by influenza: neutral *vs* disagree	1.076 (0.738–1.568)	0.582 (0.045–7.491)	1.79 (0.683–4.694)	1.436 (0.643–3.208)	1.436 (0.643–3.208)	6.859 (0.526–12,251)	2.176 (0.922–5.134)	0.414 (0.169–1.017)
Influenza vaccines are effective: agree *vs* disagree	1.023 (0.669–1.566)	1.599 (0.17–3.055)	1.222 (0.4–3.729)	1.94 (0.688–5.468)	1.94 (0.688–5.468)	7.917 (0.21–14.52)	0.938 (0.353–2.491)	0.332 (0.125–0.887)
Influenza vaccines are effective: neutral *vs* disagree	0.94 (0.654–1.352)	1.379 (0.216–8.795)	1.301 (0.557–3.039)	1.015 (0.434–2.375)	1.015 (0.434–2.375)	3.378 (0.09–6.775)	1.215 (0.51–2.895)	0.592 (0.25–1.4)
Influenza vaccines are safe: agree *vs* disagree	5.672 (3.64–8.838)***	0.228 (0.018–2.855)	7.086 (2.274–13.08)***	6.582 (2.26–10.172)***	6.582 (2.26–10.172)***	0.685 (0.008–1.365)	9.53 (2.992–18.352)***	5.513 (2.272–8.377)***
Influenza vaccines are safe: neutral *vs* disagree	2.062 (1.447–2.939)***	0.146 (0.016–1.288)	1.043 (0.471–2.314)	1.926 (0.849–4.368)	1.926 (0.849–4.368)	0.524 (0.008–1.475)	2.96 (1.183–4.407)	1.687 (0.722–3.942)
There are multiple types of influenza vaccines available: agree *vs* disagree	4.277 (2.941–6.222)***	7.818 (1.552–24.388)	9.68 (3.659–14.606)***	8.045 (3.377–13.165)***	8.045 (3.377–14.165)***	0.401 (0.009–1.394)	2.038 (0.891–4.662)	5.796 (2.317–8.499)***
There are multiple types of influenza vaccines available: neutral *vs* disagree	2.327 (1.605–3.374)***	8.178 (0.955–27.067)	3.411 (1.489–7.816)**	4.877 (2.082–7.423)***	4.877 (2.082–6.423)***	0.854 (0.016–1.539)	1.624 (0.663–3.978)	1.288 (0.512–3.239)
I plan to get an influenza vaccine the next influenza season: agree *vs* disagree	2.255 (1.492–3.408)***	6.529 (1.488–12.649)	1.841 (0.609–5.562)	0.664 (0.258–1.708)	0.664 (0.258–1.708)	12.332 (1.826–21.269)	3.695 (1.29–5.581)	3.818 (1.513–9.634)*
I plan to get an influenza vaccine the next influenza season: neutral *vs* disagree	1.918 (1.384–2.658)***	7.209 (1.174–14.28)	3.115 (1.202–8.074)	0.741 (0.33–1.665)	0.741 (0.33–1.665)	9.684 (1.984–17.265)	1.181 (0.551–2.533)	3.134 (1.511–6.499)**

*p < 0.01; **p < 0.005; ***p < 0.001.

## 4 Discussion

We found a wide range of variation in vaccine trust across Brazil, Canada, France, India, Nigeria, the United Kingdom, and the United States. Like previous global surveys on vaccine confidence (14), lower- and middle-income countries like India, Nigeria and Brazil tended to have high vaccine trust which was strongly associated with positive perspectives of COVID-19 and influenza vaccines. We found variations in the strength of these associations between countries. A country’s income level often correlates with vaccine uptake since perceptions of vaccine efficacy can vary significantly depending on the country’s economic context. Within high-income countries, elevated distrust in vaccine efficacy may be attributed to the belief in conspiracy theories, institutional distrust in vaccine administration, distribution, and marketing (7).

Our results parallel other research that shows that demographic characteristics like older age, people with no college education and those with lower incomes were less likely to have high vaccine trust (6, 18). These intersecting personal attributes that shape an individual’s perceptions are a main driving force in vaccine acceptability and likelihood of opting to get vaccinated (2). Our findings align with previous research, showcasing that individuals with greater levels of education, minimal financial hardship, and firsthand experience with COVID-19 demonstrate greater inclination to trust science, which is associated with a higher likelihood of vaccine uptake (19). Understanding these associations can aid in tailoring immunization campaigns to specific population characteristics and needs For example, understanding vaccine trust in older age groups may help to better tailor immunization programs to older adult populations who may be at higher risk of being hospitalized if they contracted COVID-19, influenza or other respiratory illnesses (20). Given that populations exhibit varying levels of trust in vaccines, when factoring in dimensions such as race, ethnicity, political affiliation, and religion, it is essential to recognize the heterogeneity within these groups (21). The diverse intersections of identities within these subpopulation can result in a variety of perspectives on vaccination, further underscoring the importance for tailored interventions that address the specific nuances of these communities.

Data suggests that intent to get a COVID-19 booster vaccine decreased from 87.9 to 71.6% in 2023 which is a cause for concern across the globe (17). However, the same study also showed that about 60% of people are more willing to get vaccinated for other non-COVID-19 vaccines due to their experiences throughout the pandemic (17). Our study showed that a higher level of concern about influenza, a strong belief in the effectiveness and safety of influenza vaccines, awareness of multiple vaccine types, and intention to get vaccinated are all associated with significantly higher odds of having high vaccine trust. A low-risk perception of COVID-19 was seen within underserved communities. It’s hypothesized that navigating the COVID-19 infodemic has led to misconceptions, and negative attitudes toward vaccination which have impacted underserved communities (22). A lack of access to trustworthy information coupled with socio-economic challenges present within underserved communities may hinder health literacy and reduce trust in public health efforts. As a result, underserved communities are disproportionately susceptible to misinformation and less inclined to recognize the advantages associated with vaccination (23, 24).

Almost half of all respondents agreed that they trust traditional protein-based vaccines more than mRNA vaccines, and around 40% are neutral. While both mRNA and traditional vaccines have been found to be safe and efficacious for COVID-19 (25), there is still a public preference for protein-based vaccines or no preference at all. Leveraging people’s concerns about COVID-19 and influenza instead of focusing on vaccine technology has been suggested as more beneficial (26). Differences in vaccine preferences can be attributed to overall availability in vaccines within an individual’s respective country. These protein-based vaccines are more prevalent in LMICs due to mRNA vaccines requiring specific infrastructure to adhere to cold-chain protocols (27). This poses a challenge to rural areas as they may not have the capacity to utilize mRNA vaccines on a widespread scale (27). Further expanding on the educational efforts to provide supplemental information regarding mRNA vaccines to individuals residing in lower-income countries is vital in increasing overall vaccine uptake when available (28).

Our results raise crucial questions about the determinants and potential implications for public health strategies. Increasing overall vaccine trust may be the key to improving respiratory vaccine uptake (17). Instead of focusing on marketing individual respiratory vaccines, efforts spent promoting overall vaccine trust may have positive implications for improving COVID-19 and influenza vaccine trust. Utilizing community-based interventions to build mutualistic relationships between vaccine providers and their associated community can provide trust building opportunities, and result in greater rates of vaccine uptake (29). Identifying specific concerns, building trust in healthcare systems, and improving communication strategies may also contribute to fostering a positive perception of vaccines. Furthermore, lessons can be learned from countries with high vaccine trust to inform best practices and potential strategies for enhancing public confidence in vaccinations.

Our study has limitations given that the survey was taken at one point in time in August 2023, after the World Health Organization officially declared the COVID-19 pandemic “over” and does not reflect the changing landscape of vaccinations. However, the survey was conducted in all seven countries at the same time, which allows us to compare the different perspectives at the same point in time. A strength of the project was using a stratum-based sample design which resulted in a sample that best represents the entire population of each country.

## 5 Conclusion

These findings show that there are differences in vaccine trust across the world. Therefore, tailoring information to the individual context may be valuable for public health immunization programs. Additionally, we found that overall vaccine trust levels are associated with confidence in COVID-19 and influenza vaccines. By understanding variations across countries, public health officials can develop targeted and culturally sensitive messaging that enhances the likelihood of successful vaccine uptake within specific communities. These differences highlight the importance of context-specific considerations and the need for comprehensive cross-cultural analysis to refine public health strategies and interventions tailored to each country’s unique circumstances. Tailoring interventions can ultimately contribute to achieving higher vaccination rates and fostering a more resilient and responsive global health landscape.

## Data Availability

All relevant data are presented in the article/Supplementary material. Further inquiries can be directed to the corresponding author.
