# Supplementary material for: A global survey to understand general vaccine trust, COVID-19 and influenza vaccine confidence
Source: Front Public Health. 2024 Nov 20;12:1406861. doi: 10.3389/fpubh.2024.1406861 (PMC11615073; doi:10.3389/fpubh.2024.1406861)
Supplement: Supplementary file 1 [file Supplementary_file_1.pdf]

Global Survey on Vaccine Confidence
Target Countries: Canada, US, UK, France, Nigeria, Brazil, India
Languages: English, French (Canadian), French (France), Portuguese
Target Vaccines: COVID-19, Influenza

I. Demographic Questions

- What is your age?
- What is your gender? [Male, Female, Other / Non-binary]
- Education (country specific)
- Income (country specific)
- Regions (country specific)
- Healthcare worker (e.g., physician, pharmacist, nurse, community health worker, other, not a healthcare worker) [Yes, No]

II. Vaccine Trust Gauge

Thinking about different vaccines recommended to you as an adult, how much trust do you have in the following five statements?

	A great deal of trust	A lot of trust	A little trust	Not trust at all
Vaccines are safe				
Vaccines protect you from serious illness				
Vaccines are a lower risk to your health (in terms of a possible bad outcome) than the diseases they prevent				
The benefits of vaccines are greater than the known or potential risks of vaccines				
Vaccines are effective				

Thinking about vaccines in general, to what extent do you agree or disagree with the following statements:

	Strongly Agree	Somewhat Agree	Somewhat Disagree	Strongly Disagree
Vaccines are important for children to have				

Vaccines are important for adults to have				

Different people and/or organizations make vaccine recommendations for adults. For each of the organizations/groups/individuals below, please state how much trust you have in their vaccine recommendations:

	A great deal of trust	A lot of trust	A little trust	No trust at all
Recommendations by a national public health agency				
Recommendations from my healthcare provider (e.g., doctor or pharmacist)				
Recommendations from my state or local health department				
Recommendation from my employer				
Recommendations from my close friends and family				

III. Vaccine Specific Questions
a) COVID-19 Vaccine

1. Have you heard of the COVID-19 vaccine?
   - o Yes
   - o No
   - o Unsure

Please indicate if you strongly agree, somewhat agree, are unsure or have no opinion, somewhat disagree, or strongly disagree with the following statements.

	Strongly agree	Somewhat agree	Neutral/No opinion	Somewhat disagree	Strongly disagree
I am concerned about illness caused by COVID-19					
COVID-19 vaccines are effective					
COVID-19 vaccines are safe					
I trust the science behind the COVID-19 vaccines					
I will continue to get boosted for COVID-19 vaccine if it is recommended to me					
It is important that any booster vaccine I get matches the current circulating variant(s).					
I trust traditional vaccines (e.g., protein-based vaccines) more than mRNA vaccines					

b) Influenza (flu) vaccines

All questions are answered on a Likert Scale (strongly agree, somewhat agree, unsure/no opinion, somewhat disagree, strongly disagree):

1. Have you heard of the influenza (flu) vaccine?
    - o Yes
    - o No
    - o Unsure

Please indicate if you strongly agree, somewhat agree, are unsure or have no opinion, somewhat disagree, or strongly disagree with the following statements.

	Strongly agree	Somewhat agree	Neutral/No opinion	Somewhat disagree	Strongly disagree
I am concerned about illness caused by the influenza (flu) virus					
Influenza vaccines are effective					
Influenza vaccines are safe					
Influenza vaccines are important for people of all ages, not just the very young, elderly, or those with weakened immune systems					
There are multiple types of influenza vaccines available					
I plan to get an influenza vaccine the next influenza season					

IV. Impact of COVID-19 on other vaccines

Has the pandemic and your knowledge of COVID-19 vaccines changed the way you view other vaccines (human papillomavirus, meningococcal, hepatitis B, influenza, etc.)?

- Yes
- No [END]
- Unsure [END]

[If yes to previous question] How has the pandemic and your knowledge of COVID-19 vaccines changed the way you view other vaccines? Please select all that apply.

- I think these other vaccines are more important as I know how critical vaccines can be
- I have more concerns about the importance of vaccines in general to our society
- I have more concerns about the safety of the other vaccines
- I have more concerns about the efficacy of the other vaccines
- I am more knowledgeable about how vaccines work in general (e.g., what antibodies are
- and their role in the immune system)
- Other (please specify)
- Unsure
